# Real-life study of the accuracy of tools for assessing fitness to drive after non-progressive acquired brain injury: a multi-centric study protocol (PREVAC study)

**DOI:** 10.3389/fnhum.2025.1539753

**Published:** 2025-03-26

**Authors:** Clothilde Rosier, Pascale Colliot, Catherine Gabaude

**Affiliations:** ^1^Laboratory of Applied Psychology and Ergonomics (LaPEA) – UMR T7708, Université Gustave Eiffel, Paris, France; ^2^Laboratory for Study of Cognitive Mechanisms (EMC) EA 3082 / MSH LSE (USR CNRS 2005), Université Lumière Lyon, Lyon, France

**Keywords:** cognitive disorders, neuropsychological assessment, driving assessment, multiprofessional, behavioral compensation, road safety

## Abstract

Non-progressive acquired brain injury can cause cognitive and behavioral damage. These disorders may hinder the driving abilities of affected individuals, increasing crash risk. Consequently, driving license regulations have required people who suffer from brain injury to be examined by an approved doctor for their driving license to remain valid. The decree of March 28, 2022 requires that approved doctors consider elements of patients’ multiprofessional evaluation, but mentions neither the on-road driving assessment nor the neuropsychological assessment. However, these assessments are an integral part of the good practice recommendations certified by the French National Authority for Health. Practitioners in rehabilitation centers are used to applying the main recommendations despite the lack of consensus about the methods and tools used. Given these new regulations and the wide variety of real-life practices, this multicenter study aims to investigate the accuracy of tools for driving skill evaluation to guide professional practices. The cross-sectional study will investigate the sensitivity and specificity of both neuropsychological tests and an on-road assessment grid (Test Ride for Investigating Practical fitness to drive), through concordance analysis between the opinions expressed by professionals and between tools. Then, a cohort study will propose longitudinal follow-up of the drivers at 6 and 12 months in order to determine the predictive performance of the various assessments in terms of road risk, and to explore the relevance of educational support on driving habits and behavior. In this context, the quality of the decision-making process for maintaining a driving license is a major issue in limiting the road risk. As part of the measures issued by the Interministerial Road Safety Committee in 2023 aimed at “better detecting, assessing and monitoring unfitness to drive”, this study presents a challenge in terms of supporting public policies. It aims to harmonize the multiprofessional evaluation recently made mandatory, in order to better inform the approved doctor’s opinion.

## 1 Introduction

### 1.1 Brain injury and accident rates

Non-Progressive Acquired Brain Injury (npABI) affects almost 300,000 people in France every year. This term covers traumatic brain injury (TBI), stroke, cerebral anoxia, encephalitis and meningoencephalitis. These injuries can cause cognitive-behavioral and sensory-motor disorders that can restrict people’s independence, and particularly their fitness to drive, since a reduced ability to react to maintain vehicle control compromises safe driving. Cognitive-behavioral disorders thus lead to an increase in road accidents and a 2.3-fold higher road risk among people with npABI (Formisano et al., 2005). This increased risk of accident for people with npABI is observed in the short (6 months), medium (3 years) and long (10 years) term since injury (Bellagamba et al., 2020; Lundqvist et al., 2008). However, many factors influence this risk, including age, education level, time since injury, extent of injury, nature of cognitive impairments and number of years licensed. This significant inter-individual variability calls for a singular evaluation of the ability to resume driving (Bellagamba et al., 2020; Novack et al., 2010). In this context, the quality of the decision-making process for maintaining the driving license of people with npABI is a key factor in limiting road risk.

### 1.2 Regulatory framework

In order to guarantee road safety for people with npABI (as well as for other road users), regulations govern their return to driving. Since 2005, “driving license holders suffering from a medical condition incompatible with […] the maintenance of their driving license are subjected to a medical examination of their fitness to drive […].” These people must apply to a doctor approved by the prefect, who will issue an opinion on their fitness to drive. The decree of March 28, 2022 listing the medical conditions incompatible with driving requires that the approved doctor’s opinion take into account the evaluation carried out by a multidisciplinary team consisting of at least a specialist (neurologist or Physical and Rehabilitation Medicine specialist—PRM) and an occupational therapist (Légifrance, 2022). The approved doctor can also ask for an on-road driving test if he considers that a real-life situation is necessary. The publication of this decree was a major step towards taking better account of the issue of resuming driving following a npABI. Nevertheless, its application raises many questions about the practice of the professionals concerned:

- The decree does not mention the assessment of the invisible impairments of npABI (neuropsychological assessment)
- The decree does not specify the conditions under which the approved doctor should request an on-road driving assessment
- Interpretation of the multiprofessional evaluation by approved doctors often varies, probably for several reasons (e.g., lack of pathology-specific training to obtain approval, diversity of practices and thus of level of expertise concerning npABI)

### 1.3 The multiprofessional evaluation

An expert group issued good practice recommendations concerning the driving ability evaluation after npABI, which were certified in 2016 by the French National Authority for Health (Haute Autorité de Santé, HAS). These recommendations aim to standardize the decision-making process of the driving ability evaluation (Haute Autorité de Santé, 2016). This guide helps to identify, assess and support people with npABI who wish to resume driving. According to these recommendations, a multiprofessional evaluation should be carried out prior to any resumption (12th recommendation). Assessments are based on (a) visual functions, with ophthalmological evaluation recommended in the event of any doubt about an alteration of the visual pathway, (b) sensory-motor functions, with an on-Road Driving Assessment (RDA) recommended in the event of sensory-motor sequelae, and (c) cognitive functions, with the recommendation to carry out an RDA in cases of cognitive-behavioral sequelae. In practice, this multiprofessional evaluation takes place in a rehabilitation center, and should include an occupational therapy assessment, a neuropsychological assessment and an RDA with a driving instructor and a therapist. The PRM doctor integrates the medical component of this multiprofessional evaluation (requesting additional tests if necessary) and submits an advisory opinion to the approved doctor on the person’s ability to resume driving. The present field research focuses on studying the performance of the neuropsychological assessment and on-road assessment, integrated into the multiprofessional evaluation, with a view to harmonizing and improving practices.

#### 1.3.1 Neuropsychological assessment

Driving is a complex activity involving perceptive-cognitive, metacognitive and social-cognitive functions (Groeger, 2015), supported by a large cerebral network at both cortical and subcortical levels. A npABI, whatever its location and origin, may affect a driver’s mental functions, making their driving less safe for themselves and other road users. As cognitive-behavioral disorders represent a higher road risk factor (Formisano et al., 2005; Bellagamba et al., 2020; Lundqvist et al., 2008; Bivona et al., 2012), an overall neuropsychological assessment is essential to assess the efficiency of the mental functions required in a driving situation. When assessing driving ability, neuropsychologists focus mainly on attentional functions. Attentional processes enable the driver to simultaneously and rapidly process different types of information (traffic signals, vehicles, pedestrians, bicycles, etc.) of varying intensity (depending on traffic conditions, for example), often over a prolonged period of time. Sawada et al. (2019) model defines attention as a multidimensional concept, distinguishing between intensive (phasic alertness and sustained attention) and selective (selective attention and divided attention) aspects, which operate under the control of a supervisory attentional system (SAS) enabling the optimal management of attentional resources in a given situation. These attentional resources are limited (Kahneman, 1973) and particularly vulnerable to the effects of brain damage. This vulnerability undoubtedly stems from the fact that the attentional system is supported by widely distributed brain networks: fronto-parietal, pulvinar, insula… (Azouvi et al., 2017; Mathias and Wheaton, 2007). Aberrant driving behavior, which is more frequent in people with npABI, is mostly attributable to inattention errors (Bellagamba et al., 2020) and attentional test results are therefore predictive of driving ability (Mathias and Lucas, 2009). For example, spatial attention disorders, such as unilateral spatial neglect, are often considered as a reason for being unfit to drive (Akinwuntan et al., 2003) and merit a systematic investigation.

Neuropsychological assessment also targets executive functions, involved in complex situations when routine actions are no longer sufficient (e.g., reacting to an unexpected event, changing route, managing speed in traffic). Diamond (2013) takes an integrative approach to modeling the executive system, distinguishing three basic functions (working memory, inhibition, and flexibility), to which are attached three higher-level functions (reasoning, problem-solving, and planning). The executive system is supported by a widely distributed anteroposterior network in which the prefrontal cortex, which is particularly vulnerable in TBI (frontal impact), plays an essential role. As driving is a complex activity during which routines are rarely sufficient, an executive dysfunction can then be considered as a predictor of underperformance during a driving assessment (Motta et al., 2014).

The neuropsychological assessment often focuses on memory abilities. Anterograde memory (the ability to acquire new information) is a fairly controversial predictor of driving ability in the literature (Wolfe and Lehockey, 2016). However, working memory (processing and temporary retention of information required to carry out activities) is particularly involved in controlled activities such as driving, when it comes to following a route or driving in heavy traffic (e.g., Aksan et al., 2015). Barrouillet and Camos recently proposed a two-system modeling of working memory based on the TBRS (Time-Based-Resource-Sharing) cognitive model (Camos and Barrouillet, 2014). The peripheral system is composed of a declarative module providing access to information stored in long-term memory, a goal module and sensory buffers (motor, phonological, and visuo-spatial). The central system processes and maintains memory traces (from the peripheral system) in an episodic buffer. Attention plays a central role in this model, as processing and storage compete for a single, limited attentional resource that must be shared between the two components. Two fMRI (functional magnetic resonance imaging) meta-analyses have confirmed the activation of the dorsolateral prefrontal cortex and posterior parietal cortex during working memory tasks (Owen et al., 2005; Rottschy et al., 2012). As these areas are vulnerable to brain damage, a working memory disorder may impair a patient’s ability to drive. In fact, several studies have shown that good working memory skills are associated with better driving performance, such as lane changing (Ross et al., 2014; Zhang et al., 2023), concentration levels (Zhang et al., 2023) or compliance with the highway code in double-tasking situations (Broadbent et al., 2023).

Although little has been documented in the literature, a psycho-behavioral assessment enables clinicians to make hypotheses about possible behavioral disorders at the root of risky behavior. Behavioral disorders linked to a lack of control (impulsivity, road rage, urgency behaviors, etc.) are frequently observed following brain injury (Billieux et al., 2014; Rochat et al., 2010), and can lead to inappropriate decisions, actions and reactions likely to increase road risk. Social cognition refers to “the ability to construct representations about the relationships between oneself and others, and to use these representations flexibly to guide one’s social behavior” (Adolphs, 2001). It requires cognitive and affective theory of mind, enabling individuals to infer others’ mental states in order to react appropriately in a shared environment, such as a road situation. Investigating social cognition thus makes it possible to understand a driver’s ability to adapt their own behavior to that of other road users. Finally, self-awareness sheds light on the development of behavioral self-regulation mechanisms, in that subjective feelings are a good predictor of self-regulation (Ang et al., 2019). Thanks to drivers’ awareness of their own abilities, they will be able to adapt their driving behavior to make it safe despite any deficits they may have (e.g., breaks in the case of asthenia, speed reduction in the case of psychomotor slowing, avoidance of rush hours in the case of sustained attention disabilities). A study has shown that self-awareness of deficits correlates with driving ability in real-life situations (Griffen et al., 2011).

#### 1.3.2 On-road driving assessment (RDA)

Complementary to the neuropsychological assessment, the RDA is considered as the “gold standard” in multiprofessional evaluation. The ecological nature of the on-road situation provides an opportunity to assess patients’ actual driving ability, enabling the adoption of compensatory strategies in the event of cognitive sequelae.

According to Michon’s hierarchical model, driving activity calls for three levels of skills and control, whose involvement varies according to the driver’s mental workload and the time constraints of the situation (Michon, 1985). The operational level involves a high time constraint (in the order of milliseconds) and a low cognitive workload, as it involves automatic action patterns. This level provides vehicle control, requiring quick and undemanding actions from the driver, such as manipulating the car controls or positioning the vehicle in the traffic lane. The intermediate tactical level involves a slightly lower time constraint during driving (in seconds) but calls on slightly higher-level cognitive processing systems, enabling the vehicle to be maneuvered in its environment. It is involved in situations requiring the driver to implement coping strategies that take into account both the perceived environment and the driver’s own driving goals (e.g., slowing down in heavy traffic or when visibility is poor). Finally, the strategic level involves a very low time constraint (in the order of minutes or even hours) but calls on very high-level cognitive functions, mainly upstream of the driving situation. In particular, it involves advance planning (before driving) and decision-making by the driver according to both external conditions (e.g., weather, traffic conditions) and the driver’s internal dispositions (e.g., fatigue, mood, and self-assurance). For example, the driver may choose to postpone departure to avoid traffic jams.

Functional analysis during RDA highlights these different processing levels, as well as the regulatory mechanisms adopted by the driver. Fuller proposes a dynamic model of driving (Task-Capability Interface: TCI) that focuses on the interaction between driver capability and task requirements (Fuller, 2005). According to the TCI model, the concept of task difficulty homeostasis consists in implementing compensatory strategies to maintain an average level of difficulty. The driver must not lose control of the vehicle (because the task is too complex), and must not lose vigilance (because the task is too simple), by constantly adjusting driving behavior. As a complement to neuropsychological assessment, the RDA assesses, in real-life situations, the implementation of coping strategies enabling safe driving ability to be maintained despite the presence of cognitive disorders.

Although recommended by the HAS, neuropsychological assessment and RDA are not mandatory. To date, the evaluation method recommended by the good practice guide has been applied by many rehabilitation centers. However, it must be noted that the tools and methods used differ considerably from one health center to another. Neuropsychological assessment practices vary in terms of the time devoted to assessment, the functions investigated, the choice of tests and the administration conditions. Similarly, for RDA, the route taken (rural, urban, expressway…) and the assessment methods (with or without an observation grid) also vary. In addition, the roles of each professional involved in the evaluation vary from one rehabilitation center to another, as well as the methods used to transmit the assessment results. The heterogeneous nature of these practices undoubtedly makes it difficult for the approved doctor to issue an opinion on the individual’s fitness to drive. The overall aim of this study is therefore to identify the most relevant methods and tools for assessing driving ability, in order to guide professionals’ practices in rehabilitation centers. In July 2023, the French Interministerial Road Safety Committee (Comité Interministériel à la Sécurité Routière, CISR) issued measures to “better detect, assess and monitor unfitness to drive” (CISR, 2023). Against this backdrop, this study aims to support public policy by harmonizing the multiprofessional evaluation recently made mandatory, so as to better inform the approved doctor’s opinion.

## 2 Method and analysis

### 2.1 Study design and participants

The PREVAC study (driving skills assessment program) is a regional (Rhône-Alpes), multicenter, real-life prospective and exploratory study that is:

- Cross-sectional for concordance and correlation analysis between the assessment tools available at the time of the approved doctor’s opinion;
- Longitudinal for follow-up at 6 and 12 months after the approved doctor’s opinion, with data collected on participants’ driving habits and road risk.

This study is being carried out in collaboration with eight rehabilitation centers belonging to a regional network for caring and supporting people with npABI in the Auvergne-Rhône-Alpes region (Resaccel). The protocol has been designed, under the supervision of an international scientific committee, to ensure that the assessment is as standardized as possible between the healthcare centers taking part in the study (same equipment, same training, same procedure, same instructions, comparable on-road-assessment; the protocol and the case report form are available on OSF). At each center, a multiprofessional team was trained to assess the driving ability of their volunteer patients, according to the study protocol. Each team comprises a PRM doctor, a psychologist specialized in neuropsychology, an occupational therapist and a driving instructor. Within each rehabilitation center, an investigator monitors the study. Participants do not make the journey specifically for the study but as part of the follow-up offered to them by the centers. From there, they travel to the center using their usual means of transport.

Each rehabilitation center will recruit around 10 to 20 patients, up to a total of 100 participants. A post hoc sensitivity analysis was performed using G*Power Software Version 3.1.9.7 (Faul et al., 2007) to determine the minimum effect size that could be achieved with 100 participants. A post hoc power analysis for an independent-sample t-test was conducted assuming one-tailed testing. For effect size varying from large (d = 0.8) to small (d = 0.2), 80% power and alpha error probability of α = 0.05 a sample size interval of at least 12 to 156 participants is suggested. With a sample of 100 participants to be included in this study and 80% power the effect size is estimated at 0.25. Each participant must have a npABI and be between the ages of 21 and 65, in order to avoid confusing the issues of ageing and non-progressive acquired brain injury, people over 65 were excluded from the study and treated at their center for routine care. All participants must have held a Category B driving license (light vehicles) for at least 3 years and have driven a minimum of 10,000 km, so as not to assess novice drivers. They have been examined by the PRM doctor, who confirms that there are no contraindications to driving other than the pathology under study. They also gave their consent to take part in the PREVAC study. The ethical committee has classified this research as a non-interventional, involving no risk or constraint for participants, and in which all procedures are carried out in the usual way. They must not have a medical condition (other than npABI) that is incompatible with maintaining their driving license, according to the list of conditions mentioned in the decree of March 28, 2022. They must not have any oral comprehension disorders that can be perceived by the therapist, must not be pregnant (to avoid an interruption of pregnancy in the event of an accident and the resulting psycho-social and legal risks), and must not be under the influence of any Level 3 medication that could present a driving risk.

### 2.2 Study protocol

#### 2.2.1 Cross-sectional study

Each participant’s driving ability is assessed by experienced field assessors as follows:

##### 2.2.1.1 Consultation with a PRM doctor

The purpose of this medical consultation is to check the inclusion/non-inclusion criteria and define the pathology(ies) and list the medication prescribed and taken by the participant, in order to study their possible influence on driving ability. These sensitive data are not investigated in the study but may be partially mentioned in the final multiprofessional opinion.

##### 2.2.1.2 Neuropsychological assessment

This assessment is carried out by a psychologist specialized in neuropsychology. It includes a series of validated standardized tests investigating attentional, executive and psycho-behavioral functions. The list of tests was co-constructed according to different criteria:

- psychometric qualities (sensitivity, validity, reliability)
- relevance established in the literature in relation to driving ability evaluation
- availability and frequency of use in current practice (assessed through a survey of rehabilitation centers)
- administration conditions (essentially non-verbal tests for use with patients with expressive aphasia; non-bimanual tests allowing inclusion of patients with hemicorporeal deficits; duration deemed acceptable and equipment easy to obtain and use).

The neuropsychological assessment lasts around 3 h, divided into two one-and-a-half-hour sessions, each with a five- to ten-minute break. The order of administration of the tests was established so as to alternate tasks according to their format (computerized vs. paper-and-pencil), their mental load (higher or lower), their sensitivity to fatigue, and the cognitive functions called upon. The standardized neuropsychological assessment protocol is described in the case report form. It includes the following tests: Alertness (TAP 2.3.1), Divided attention (TAP 2.3.1), Epworth scale, Trail-Making-Test (TMT), Go/Nogo (TAP 2.3.1), Basic Empathy Scale in Adults (BES-A), Neglect with central task (TAP 2.3.1), Tour of London (TOLDX 2^nd^ edition), Working memory difficulty level 3 (TAP 2.3.1), Sustained attention color-or-form version (TAP 2.3.1) and UPPS Impulsive Behavior Scale. Lastly, the Brain Injury Driving Self-Awareness Measure—BIDSAM (Gooden et al., 2017) is part of the neuropsychological assessment, but will be completed following the RDA.

For each test, the examiner collects raw and standardized scores (each participant’s performance is compared to the standard group). Based on this assessment, the neuropsychologist then issues an opinion (favorable, unfavorable or reserved) on the estimated compatibility between the participant’s cognitive functions and the return to driving.

##### 2.2.1.3 Occupational therapy assessment

The occupational therapist carries out this assessment according to a non-standardized protocol, in order to investigate the participant’s sensory-motor functions, and to consider possible vehicle adaptations. His role is mainly to determine the necessary adaptation of the participant’s car and, thanks to the pluriprofessional advice given at the end of the process, to pass on his opinion to the approved doctor. The occupational therapist also administers questionnaires to obtain information on the participant’s driving before brain injury: Driving Habits Questionnaire (DHQ), Driving Behavior Questionnaire (DBQ) and perceived road risk.

##### 2.2.1.4 On-road driving assessment (RDA)

A driving instructor and a therapist carry out this 45-min assessment in a dual-control driving school car. In order to limit variability linked to the geographical diversity of the rehabilitation centers, driving routes were standardized between centers to ensure that they were comparable in terms of the traffic and road situations encountered. This route standardization was established in accordance with recommendations certified by the HAS (Haute Autorité de Santé, 2016). The TRIP (Test Ride for Investigation Practical fitness to drive) observation grid and scoring system are detailed in the case report form. Quantified measures of driving performance are collected during the assessment (15 sub-scores and a total score out of 100). The professionals present in the vehicle fill in this grid jointly after the RDA. The main interest of this grid, and the reason for its choice in this study, lies in the relevance of the tactical and strategic compensation scores in relation to Michon’s (1985) model. These scores reflect the behavioral self-regulation mechanisms adopted by drivers in order to compensate for any cognitive disorders and guarantee homeostasis of the task difficulty as described in the TCI model. Following this assessment, professionals issue an opinion on the participant’s driving ability (favorable, unfavorable or reserved).

##### 2.2.1.5 Multiprofessional synthesis

Professionals then work together to issue a joint opinion (favorable, unfavorable or reserved) on the participant’s driving ability, based on the assessments carried out.

##### 2.2.1.6 Patient feedback session

Professionals communicate the multiprofessional opinion to the participant, if possible in the presence of one or more relatives. They then provide the participant with documents required for the approved doctor visit: list of approved doctors, multiprofessional evaluation report, medical questionnaire, cerfa 14880*02 and, if necessary, a tutorial on completing formalities with the French National Agency for Secure Documents (Agence Nationale des Titres Sécurisés, ANTS).

##### 2.2.1.7 Approved doctor visit

Following the multiprofessional evaluation, participants then have to undergo a consultation with an approved doctor from their department. They then inform the study investigator of the opinion issued by the approved doctor (fitness, temporary fitness and/or with restriction(s), unfitness).

##### 2.2.1.8 Participant support

In the event of a favorable opinion, professionals help the participant with administrative formalities (ANTS website, road safety education office in the event of changes to the driving cab). In the event of an unfavorable or reserved opinion, professionals propose solutions to the participant (driving restrictions, rehabilitation in a simulator or driving school, time lapse before a new assessment, alternatives to driving, etc.).

#### 2.2.2 Longitudinal study

Six and 12 months after deliberation of the approved doctor’s opinion, the study investigators of the rehabilitation centers will send questionnaires to each participant of the cross-sectional study in order to study the road risk prediction performance of the tools and professional opinions. These questionnaires can also be completed with the help of a third party or a therapist. They will aim to gather information about participants’ driving in the 6 and 12 months since the opinion on fitness to drive was delivered. The elements investigated will concern:

- aberrant driving behavior with DBQ validated in French (Guého et al., 2014),
- possible changes in driving habits with DHQ, based on the Canadian Jerome Driving Questionnaire (JDQ©), and
- road risk (accident rate and perceived risk).

Six months after the approved doctor’s opinion, participants will also be asked to complete a questionnaire (support for resuming driving questionnaire) to assess the relevance of multiprofessional educational support aimed at encouraging them to resume driving or dissuading them from doing so. In order to limit participant drop-outs, if they do not respond within one month, a reminder is sent by their SMR referent, with a one-month response period and this procedure will be repeated a second time if no response is received after this deadline. After these two reminders, the SMR referent may also contact the participant by phone to ensure that the e-mails have been received. If the participant refuses or does not reply, the SMR referent sends a final e-mail to the participant to inform them that they will be excluded from the longitudinal study.

### 2.3 Analysis

Participant characteristics and assets will be described and compared. Reasons for refusing to participate will be collected. Descriptive statistics on participant characteristics will be presented. The characteristics of participants included will be compared with those of non-included participants in the eligibility register to ensure their representativeness. Quantitative variables will be described in terms of mean, standard deviation, median, interquartile range and extreme values. Qualitative variables will be described in terms of absolute frequency and percentage per modality. The alpha threshold for statistical significance is set at 5%; 95% confidence intervals will be presented. All the variables collected for people included in the study will be described. Missing data will not be replaced in this study and some participants might be secondary excluded from certain analyses. Sub-group analyses may be carried out, if statistically robust, depending on the number of fitted cars required and the conclusion of the medical examination.

#### 2.3.1 Cross-sectional study

##### 2.3.1.1 Main criterion: concordance between professional assessments

To meet the main objective, the concordance of the results of the neuropsychological assessment, RDA and multiprofessional evaluation will be studied in pairs (agreement between professional assessments). Each professional involved in the study will give a “favorable,” “reserved” or “unfavorable” opinion based on their practice. Evaluation criteria will correspond to cross-tabulated numerical values of results from each assessment tool studied (neuropsychological assessment, RDA and multiprofessional evaluation). “Reserved” opinions indicating driving restrictions will initially be classified as “unfavorable,” so that the results can be expressed as a binary Favorable/Unfavorable variable for each assessment. The RDA results will also include the number of driving instructor interventions on pedals, steering wheel and other car controls, using categorical variables. The values *a*, *b*, *c* and *d* (Table 1) will be used to perform the concordance test detailed in the next section (“Statistical analysis”).

**Table 1:** Evaluation criterion of the main objective for contingency table.

		Assessment x_i_	
		Favorable	Unfavorable
Assessment x_ii_	Favorable	*a*	*b*
	Unfavorable	*c*	*d*

##### 2.3.1.2 Secondary evaluation criteria (criteria A and B)

A cross-sectional analysis of professional evaluations will be performed to meet the following secondary objectives:

- Study the concordance between each professional assessment (cf. main objective) and the approved doctor’s opinion concerning the participant’s ability to resume driving;
- Study the concordance between professional assessments (cf. main objective) and the TRIP

###### 2.3.1.2.1 Secondary criterion A: concordance professional assessment / approved doctor’s opinion

Evaluation criteria for secondary objective A will be those used for the main objective, crossed with those of the approved doctor’s opinion, expressed as a binary variable Fit / Unfit to resume driving (Table 2).

**Table 2:** Evaluation criteria of the secondary objective A for contingency table.

		Assessment x_i_	
		Favorable	Unfavorable
Approved doctor’s opinion	Fit	*a*	*b*
	Unfit	*c*	*d*

###### 2.3.1.2.2 Secondary criterion B: concordance professional assessment / TRIP grid

An analysis identical to the main objective will be carried out, cross-referencing the results of the professional assessments with that of the TRIP, expressed as a binary variable Favorable / Unfavorable (Table 3).

**Table 3:** Evaluation criteria of the secondary objective B for contingency table.

		Assessment x_i_	
		Favorable	Unfavorable
TRIP grid	Favorable	*a*	*b*
	Unfavorable	*c*	*d*

The TRIP score will also be analyzed continuously (total adjusted score out of 100), in total and by sub-score. Correlation analysis will also be carried out between the raw results from the neuropsychological tests and the TRIP grid, in order to identify the neuropsychological tools most predictive of driving ability in real-life situations.

#### 2.3.2 Longitudinal study (secondary evaluation criteria C to E)

Six and 12 months after deliberation of the approved doctor’s opinion, a longitudinal analysis will enable the following secondary objectives to be met:

C. Estimate the road risk prediction performance metrics of each assessment tool (cf. main objective) and of the TRIP, as well as of the approved doctor’s opinion;
D. Explore participants’ changes in their driving habits;
E. Explore the impact of educational support to encourage or dissuade driving resumption (only explored after 6 months).

##### 2.3.2.1 Secondary criterion C: road risk performance metrics 6 and 12 months after deliberation of the approved doctor’s opinion

The predictive performance indicators of assessment tools, TRIP grid, and approved doctor’s opinion will be estimated in relation to the road risk perceived by participants 6 and 12 months after deliberation of the approved doctor’s opinion (cf. secondary criterion D). The patient will indicate the increase in road risk to road risk according to their perception, in the form of a ternary variable in response to the question “How would you assess your road risk”: (i) lower than before, (ii) identical to before, (iii) higher than before the brain injury. The condition evaluated will be absence of increased road risk perceived by participants 6 and 12 months after the approved doctor’s opinion (negative result), corresponding to the “favorable” modality for assessments carried out in rehabilitation centers (neuropsychological, RDA and multi-professional evaluation) and “fit” for the approved doctor’s opinion (Table 4).

**Table 4:** Confusion matrix.

		Reality: perceived increased road risk 6 and 12 months after the approved doctor’s opinion has been issued	
		No increased road risk	Increased road risk
Prediction: assessment x_i_	Favorable	Real negative (RN)	False negative (FN)
	Unfavorable	False positive (FP)	Real positive (RP)

The TRIP score will also be analyzed continuously (total adjusted score out of 100), in total and by sub-score.

##### 2.3.2.2 Secondary criterion D: changes in driving habits

Based on the results of the DHQ and DBQ questionnaires, changes in driving habits will be assessed using the difference scores between “before npABI” and M6 and M12 after the approved doctor’s opinion. These difference scores can be calculated on the basis of responses to each questionnaire item, as well as on the basis of each investigated dimension by grouping items (speed, duration, frequency, etc.).

##### 2.3.2.3 Secondary criterion E: impact of pedagogical support

In the context of pedagogical support, the usefulness of the advice given will be analyzed using a 5-point Likert scale, ranging from “Very useful” to “Not at all useful.” The information can be collected globally, or by type of advice.

#### 2.3.3 Statistical analysis

To meet the main and secondary A and B objectives, concordance tests will be used to verify that two independent abilities to resume driving measures (two tests) of the same modality—“Favorable” or “Unfavorable”—are equal. Concordance analyses between tests (binary variables) will be expressed in the form of concordance percentages, simple kappa coefficient and its 95% confidence interval. Analyses between TRIP (continuous variable) and other tests (binary variables) will be conducted using *t*-tests or Mann–Whitney tests depending on the TRIP score distribution. Multivariate regression will also be used to study measures’ concordance and prediction levels, for both objectives. It will be logistic or linear, depending on the studied variable (i.e., approved doctor’s opinion for objective A and TRIP results for objective B).

A sensitivity and specificity analysis of the approved doctor’s opinion will also be carried out. Performance metrics (Secondary Objective C) will be calculated from the confusion matrix values (Table 4):

$$\mathrm{Accuracy:}\frac{RP+RN}{RP+RN+FP+FN}$$

$$\mathrm{Sensitivity (Se):}\frac{RP}{RP+FN\text{.}}$$

$$\mathrm{Specificity (Sp):}\frac{RN}{FP+RN}$$

$$\mathrm{Positive predictive value (PPV) or Precision (Pr):}\frac{RP}{RP+FP}$$

$$\mathrm{Negative predictive value (NPV):}\frac{RN}{FN+RN}$$

### 2.4 Expected results

As this is an exploratory study, no assumptions are made concerning concordances between the different professional opinions. However, in light of the literature concerning coping strategies used by people with npABI, we can expect some results:

In the event of an unfavorable opinion on the neuropsychological assessment and a favorable opinion on RDA, we should observe high compensation scores on the TRIP, as well as a low score on the BIDSAM, indicating good metacognition enabling the driver to adapt their driving to their difficulties.

In the event of an unfavorable opinion on both the neuropsychological assessment and RDA, we should observe low compensation scores on the TRIP and a high score on the BIDSAM, indicating a low awareness of difficulties that does not allow the use of compensatory strategies.

TRIP compensation scores and the BIDSAM score should be good predictors of road risk.

## 3 Discussion

Driving activity is associated with greater community participation, better functional outcomes, fewer symptoms of depression, and greater life satisfaction (Novack et al., 2021). For these reasons, resuming driving represents a major challenge for people with npABI. However, in order to limit the increased road risk to which this population is exposed (Formisano et al., 2005; Bellagamba et al., 2020; Lundqvist et al., 2008), regulations require them to undergo a medical examination (Légifrance, 2022). Although the HAS recommendation (Haute Autorité de Santé, 2016) guidelines the multiprofessional evaluation, the decision-making process for maintaining the driving license of people with npABI has yet to be defined. This study will underpin good practice recommendations certified by the HAS, which are currently based on expert agreement. By gathering scientific evidence, this study aims to standardize the evaluation and support process for people with npABI who need to resume driving.

### 3.1 Cross-sectional study

Thanks to the development of a multiprofessional evaluation protocol based on HAS recommendations, results will support the relevance of assessments recommended by the experts, and help refine their practical application (tools and methods). Concordance analysis will clarify the relevance of each assessment (neuropsychological, on-road and multiprofessional).

#### 3.1.1 Relevance of the neuropsychological assessment

- In the event of concordance between the neuropsychologist’s opinion and other assessments (RDA, multiprofessional, approved doctor’s opinion), the neuropsychological assessment can then be considered to be a good predictor of driving ability, particularly by shedding light on the driving restrictions to be recommended.
- In the event of a discrepancy between the neuropsychologist’s opinion and other assessments (RDA, multiprofessional, approved doctor’s opinion), the neuropsychological assessment cannot be considered a reliable predictor of driving ability, in line with the mixed findings reported in the literature (Wolfe and Lehockey, 2016; McKay et al., 2016; Ortoleva et al., 2012). However, this assessment will still be of obvious interest for detecting any reduction in useful field of view, raising awareness of certain deficits, or suggesting rehabilitation options adapted to the person’s cognitive profile. The neuropsychologist’s role is decisive in the project to resume driving (Perna et al., 2021).
- Neuropsychological tests whose scores are significantly correlated with the TRIP score can be considered the most predictive of driving ability. These results will help guide the choice of neuropsychological tools.

#### 3.1.2 Relevance of on-road assessment

- In the event of concordance between opinion following RDA and other assessments (neuropsychological, multiprofessional, approved doctor’s opinion), the assessment tools mentioned can be considered to be good predictors of driving ability, in accordance with the literature (Marshall et al., 2007).
- In the event of a discrepancy between RDA and other assessments, the RDA value will remain undeniable, but results can be explored and discussed depending on which assessment there is a discrepancy with, and the direction of this discrepancy. More specifically, a discrepancy between RDA and neuropsychological assessment can highlight coping strategies linked to awareness of cognitive disorders. In fact, correlation between neuropsychological assessment and actual driving may depend on the individual’s level of awareness of their abilities (Griffen et al., 2011). These results would not, therefore, call into question value of the RDA. On the other hand, a discrepancy between the opinion given following RDA and that given by the PRM doctor (multiprofessional evaluation) or the approved doctor may raise questions about value of the RDA, or at least the importance attached to it by doctors (PRM or approved doctor).
- In the event of concordance between the TRIP score and the various assessments, TRIP can be considered a relevant tool for predicting driving ability. Conversely, in the event of discrepancies between TRIP and other assessments, the usefulness of this grid can be discussed. Further analysis could highlight the relevance of certain sub-scores.
- In the event of concordance between the number of interventions on the controls and professional opinions, this criterion can then be considered to be a good predictor of driving ability. On the other hand, in the event of discrepancies, a certain tolerance may be accepted if the driving instructor intervenes on the controls during RDA.

#### 3.1.3 Relevance of multiprofessional evaluation

- Agreement between the multiprofessional opinion and that of the approved doctor will enable the approved doctor to get additional information, in line with this evaluation recommended by the HAS and now mandatory.
- A discrepancy between the multiprofessional opinion and that of the approved doctor will help to identify situations for which an opinion is difficult to formulate. Using the scientific evidence gathered, an analysis of advantages and disadvantages can be carried out to shed more light on the fitness to drive evaluation, with a view to helping approved doctors identify the best tools for deliberating their opinions.

### 3.2 Longitudinal study

Thanks to the longitudinal study, further analyses will enable us to measure the relevance of the multiprofessional evaluation by comparing each assessment with the road risk. Assessments that are significantly correlated with perceived road risk (as well as with self-reported accident rates) can be considered as good predictors of road risk, further supporting their relevance.

The longitudinal study will also shed light on any changes in driving habits adopted by participants since regularization of their driving license. These analyses will quantify the adoption of coping strategies related to npABI sequelae. Complementary analyses would also enable us to study relationships between changes in driving habits and various assessment scores. For example, a high correlation between changes in driving habits and BIDSAM would highlight the link between metacognition and behavioral regulation (adjusting driving to compensate for cognitive sequelae).

Lastly, this longitudinal study will provide some answers to the HAS recommendation that people with npABI be given the best possible support to resume or stop driving. By questioning participants, the support for resuming driving questionnaire will highlight the self-reported relevance of multiprofessional assessment for:

- Judging their driving ability
- Becoming aware of their driving ability
- Obtaining information and advice from the team
- Adjusting their driving (in the case of unrestricted fitness to drive)
- Understanding the reasons for unfitness to drive or driving restrictions (in the case of fitness with restriction codes)
- Dissuading them from driving (in the event of unfitness)

### 3.3 PREVAC study limits

Brain injury criteria are not controlled in the study, lesion location, severity and extent were not identified, suggesting heterogeneous clinical profiles. In addition, the temporal distance of the lesion was collected but not delimited. Although cognitive sequelae may persist for many years after brain injury, they tend to diminish over time. What’s more, the temporal distance of the lesion favors the development of compensatory strategies.

These factors generate a diversity of cognitive-behavioral profiles that contribute to a lack of homogeneity in the population studied, complicating interpretation of the results. It could be difficult to identify the profiles most likely to resume driving, and the study’s conclusions would then not be applicable to the population as a whole. Secondary analysis with subgroup studies will be conducted to measure differences linked to the temporal distance of the lesion.

### 3.4 PREVAC study outlooks

#### 3.4.1 Standardizing professional practices

Based on conclusive results of this study, a parallel action-research project is being carried out at regional level, with the aim of standardizing real-life practices and make it easier to write pluriprofessional advice. Based on focus groups conducted within rehabilitation centers, the cross-sectional study protocol will be improved to meet everyone’s interests. These regional outlooks can then be extended to the rest of French metropolitan and even overseas departments.

This study could also pave the way for future research using randomized controlled trials to verify the relevance of the assessments and tools. For example, in order to highlight the value of neuropsychological and on-road assessments (currently not required by law), it might be interesting to compare the road risk of a group of patients who have undergone these assessments, versus a group of patients who have only undergone a sensory-motor and medical assessment.

#### 3.4.2 Public policy support

Since the publication of the decree of March 28, 2022, people with npABI have been required to undergo a multiprofessional evaluation, so that the approved doctor can issue an informed opinion on their driving ability. In the context of these recent regulations, this multicenter study presents an opportunity to support public policy by shedding new light on the French Road Safety Delegation (Délégation à la Sécurité Routière, DSR).

Axis 2 of the July 17, 2023 of the CISR aims to ‘better detect, assess and monitor unfitness to drive’. More specifically, the seventh measure raised by this committee is to improve the medical examination of fitness to drive. This measure focuses on reinforcing specialized technical platforms to better assess the medical fitness to drive of patients with cognitive and neuromotor disorders. In this context, depending on the results obtained, this study could clarify the value of the multiprofessional assessment for the approved doctor. It would provide scientific evidence to support the consideration of various assessments carried out in current practice. In particular, as neuropsychological assessment is not included in the decree, this study aims to take better account of invisible disabilities during fitness to drive medical examinations. To achieve this, we hope to identify the best-performing tools that are the most predictive of actual driving ability, considering drivers’ ability to compensate for their cognitive disorders.

The CISR also recommended a measure to reinforce the training of approved doctors in terms of the medical content of their examinations. The operational outcomes of this study will consist in disseminating its conclusive results to professionals concerned by this fitness to drive medical examination. Documentation can be produced for use by teams at rehabilitation centers and approved doctors. With the financial support of the DSR, which funds the PREVAC project, key elements of the study could be integrated into the training of approved doctors, and could clarify the decree of March 28, 2022.
